# A method for improving semantic segmentation using thermographic images in infants

**DOI:** 10.1186/s12880-021-00730-0

**Published:** 2022-01-03

**Authors:** Hidetsugu Asano, Eiji Hirakawa, Hayato Hayashi, Keisuke Hamada, Yuto Asayama, Masaaki Oohashi, Akira Uchiyama, Teruo Higashino

**Affiliations:** 1 Technical Department, Atom Medical Corporation, 2-2-1, Dojo, Sakura-ku, Saitama city, Saitama 338-0835 Japan; 2 Department of Neonatology, Nagasaki Harbor Medical Center, 6-39, Shinchi-machi, Nagasaki City, Nagasaki 850-8555 Japan; 3 Department of Neonatology, Kagoshima City Hospital, 37-1 Uearata-cho, Kagoshima City, Kagoshima 890-8760 Japan; 4 Department of Clinical Engineering, Nagasaki Harbor Medical Center, 6-39, Shinchi-machi, Nagasaki City, Nagasaki 850-8555 Japan; 5 Department of Comprehensive Community Care Education, Nagasaki University Graduate School of Biomedical Sciences, 1-14, Bunkyo-machi, Nagasaki City, Nagasaki 852-8521 Japan; 6 Mobile Computing Laboratory, Graduate School of Information Science and Technology, Osaka University, 1-5, Yamadaoka, Suita, Osaka 565-0871 Japan

**Keywords:** Thermography, Semantic segmentation, Infants, Temperature

## Abstract

**Background:**

Regulation of temperature is clinically important in the care of neonates because it has a significant impact on prognosis. Although probes that make contact with the skin are widely used to monitor temperature and provide spot central and peripheral temperature information, they do not provide details of the temperature distribution around the body. Although it is possible to obtain detailed temperature distributions using multiple probes, this is not clinically practical. Thermographic techniques have been reported for measurement of temperature distribution in infants. However, as these methods require manual selection of the regions of interest (ROIs), they are not suitable for introduction into clinical settings in hospitals. Here, we describe a method for segmentation of thermal images that enables continuous quantitative contactless monitoring of the temperature distribution over the whole body of neonates.

**Methods:**

The semantic segmentation method, U-Net, was applied to thermal images of infants. The optimal combination of Weight Normalization, Group Normalization, and Flexible Rectified Linear Unit (FReLU) was evaluated. U-Net Generative Adversarial Network (U-Net GAN) was applied to thermal images, and a Self-Attention (SA) module was finally applied to U-Net GAN (U-Net GAN + SA) to improve precision. The semantic segmentation performance of these methods was evaluated.

**Results:**

The optimal semantic segmentation performance was obtained with application of FReLU and Group Normalization to U-Net, showing accuracy of 92.9% and Mean Intersection over Union (mIoU) of 64.5%. U-Net GAN improved the performance, yielding accuracy of 93.3% and mIoU of 66.9%, and U-Net GAN + SA showed further improvement with accuracy of 93.5% and mIoU of 70.4%.

**Conclusions:**

FReLU and Group Normalization are appropriate semantic segmentation methods for application to neonatal thermal images. U-Net GAN and U-Net GAN + SA significantly improved the mIoU of segmentation.

## Background

Neonatal body temperature is known to have a significant effect on prognosis [1–5], and body temperature is inversely correlated with mortality in infants [1, 2, 4]. As temperature management is clinically important in neonatal care, a number of organizations, including the World Health Organization (WHO), have proposed guidelines for neonatal temperature management [6–9]. However, there is still a lack of evidence regarding the optimal body temperature for infants [8]. Karlsson et al. [10] investigated the differences in temperature of the head, body, arms, legs, and feet of healthy infants, and reported that differences in skin temperature at different sites can be used for diagnosis of infants [10–15]. Knobel et al. [15] measured body temperature using thermistors attached to the abdomen and feet of very low birth weight (VLBW) infants, and reported its relation to peripheral vasoconstriction. These reports suggest the importance of temperature control and detailed regional temperature measurement in infants. However, these studies used contact-type probes, which are associated with a number of issues that lead to inaccuracy of measurements, including probe position, fixation method, contact with the skin, and the inability to measure the temperature distribution over the whole body. Therefore, a number of recent studies used infrared thermography, a non-contact, continuous thermal imaging technique that uses infrared light emitted from objects in accordance with heat, which is assumed to be the surface temperature in neonates [16–21]. At present, contact-type probes are used for continuous temperature measurement, but their use is associated with hygiene risks and they can damage the fragile skin of infants. However, there is increasing interest in the application of neonatal thermography as it can reduce these risks. Medical adhesive-related skin injuries (MARSI) are a known clinical problem, which is particularly important in neonatal care, and the risk of such injuries must be reduced [22–24]. Knobel et al. [16] examined the differences in temperature distribution between the chest and abdomen due to necrotizing enterocolitis (NEC) in VLBW infants, and reported that children with NEC had significantly lower abdominal temperatures compared to healthy infants. Using thermal imaging, Knobel et al. [17] also demonstrated that the temperature of the feet was higher than that of the abdomen within the first 12 h of life in VLBW infants. Abbas et al. [18] developed a detailed measurement model to accurately measure body temperature in infants based on thermal images, and Ussat et al. [19] proposed a non-contact method for measurement of respiratory rate based on the temperature difference of inhaled air.

Therefore, there have been a number of studies on the utility of thermography for monitoring the body temperature of infants. However, it was necessary to set the region of interest (ROI) manually for each analysis, preventing continuous evaluation and therefore the evaluation was not strictly quantitative.

To address this issue, there have been a number of studies regarding automated processing of ROIs by computer. Duarte et al. [25] and Rodriguez et al. [26] used image processing methods, such as edge extraction and ellipse fitting, for automatic ROI extraction in thermal images of adults. However, these methods aim to exclude other regions from the ROI, and are unable to segment the human body into regions. Abbas et al. [27] proposed a method for tracking analysis points using temporally continuous thermal images of infants, which allowed analysis of the temporal variability of the analysis points. However, it was still necessary to set the analysis points manually in their method.

Deep Learning may be applicable to address the disadvantages of these methods. There has been significant progress in research on semantic segmentation, especially in the field of automatic driving [28–30]. The application of semantic segmentation to thermal images of infants would allow detailed analysis of global information. Ronneberger et al. [31] proposed U-Net as a segmentation method for cellular images. U-Net has been used for segmentation of biomedical images, and has been applied in a number of studies because of its stability and high performance. Antink et al. [32] proposed a method for segmenting the body parts of neonates from RGB images. In addition, there have been a number of studies on automatic classification of organs on magnetic resonance imaging (MRI) and computed tomography (CT) images [33–35]. Deep Learning has also been applied to thermal images for medical applications. Lyra et al. [36] applied Yolov4 [37] to thermal images for automatic extraction of patients and medical staff and calculation of vital signs from the detected regions. Kwasniewska et al. [38] performed image resolution enhancement of thermal images to increase the accuracy of estimation of vital signs from thermal images. Moreover, Ekici et al. [39] applied Deep Learning to detect breast cancer in thermal images. However, the application of Deep Learning to thermal images in neonates has not been investigated in sufficient detail.

Generative Adversarial Network (GAN) is a Deep Learning method that has been under development in recent years. GAN is a learning method proposed by Goodfellow et al. [40] in which a Generator network that generates images and a Discriminator network that determines whether an input image is a natural or generated image compete with each other. There have been a number of reports of the application of GAN in image style transformation, etc. [41, 42]. It has been applied in a number of fields, including Semantic segmentation, where the loss function is difficult to define. Self-Attention (SA) [43] is a method that has had a significant impact on improving the performance of Deep Learning. There has been marked progress in the development of Deep Learning in the field of natural language processing, and high-performance networks using the Attention mechanism have been proposed [44, 45]. SA is a method that applies these techniques to image processing, enabling more complex analysis by learning and assigning meaning to relationships between pixels, such as between words in a sentence. In conventional convolutional networks, local variations in an image are extracted and weighted to achieve detection. SA takes into account the relations between the intensities of the pixel values in weighting, making it possible to express changes in the importance of pixel values.

For continuous quantitative analysis of thermal images, semantic segmentation can be applied for automatic ROI setting in infants. In this study, we propose a suitable method for semantic segmentation of thermal images in infants. An accurate semantic segmentation method would enable detailed analysis of the temperature of each region of an infant’s entire body surface. This will enable early detection of diseases, such as sepsis and NEC, which are currently difficult to detect. Early detection of these diseases will lead to better prognosis and to new standards of care. Considering the extension to disease prediction using Deep Learning, we investigated methods of segmentation with the maximum possible accuracy and detail. The methods and their performance were evaluated using thermal images acquired in a clinical setting.

## Methods

Twelve preterm infants without congenital or underlying diseases, born at Nagasaki Harbor Medical Center (NHMC) and requiring incubator support, were included in this study. The characteristics of the patients are shown in Table 1. The median ± standard deviation (SD) of the gestational age of the infants included in the study was 34 ± 2.8 weeks, birth weight was 2053 ± 712 g, mean age at the start of imaging was 0 + 0.8 days, and male:female ratio was 7:5. This study was approved by the Ethics Committee of Nagasaki Harbor Medical Center (Approval No. NIRB No. R02-006). The research was carried out in accordance with the Declaration of Helsinki.

**Table.1:** Participant characteristics

Characteristic (*n* = 12)	Median ± SD
Gestational week at delivery	34 ± 2.8
Birth weight (kg)	2053 ± 712
Age (days)	0 ± 0.8
Sex (male)	7 (58%)

**Fig. 1:**
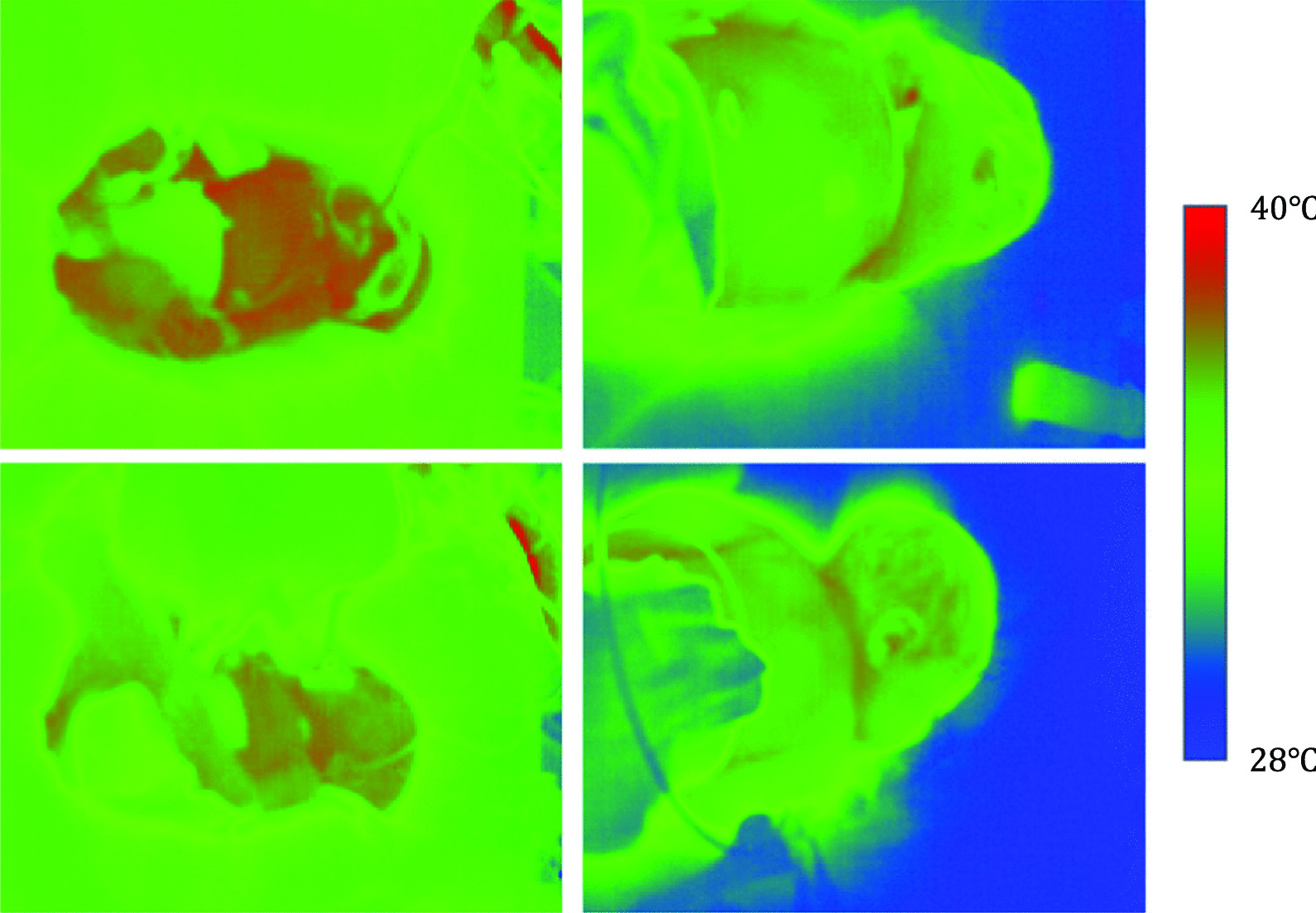
Thermographic images. Many variations in thermal images were obtained with different sizes and positions of the infants: blue, 28 °C; red, 40 °C

A thermography camera was installed on the upper part of the incubator at the side closest to the feet of the infant. Data with a resolution of 320 × 256 were acquired at 1 fps using a thermal camera (FLIR A35; FLIR, Middletown, NY, USA). Thermographic images with various variations in size, position, etc., were captured for 66–140 h in each case, for a total of 1032 h. Figure 1 shows an example of a thermal image obtained using this system.

**Fig. 2:**
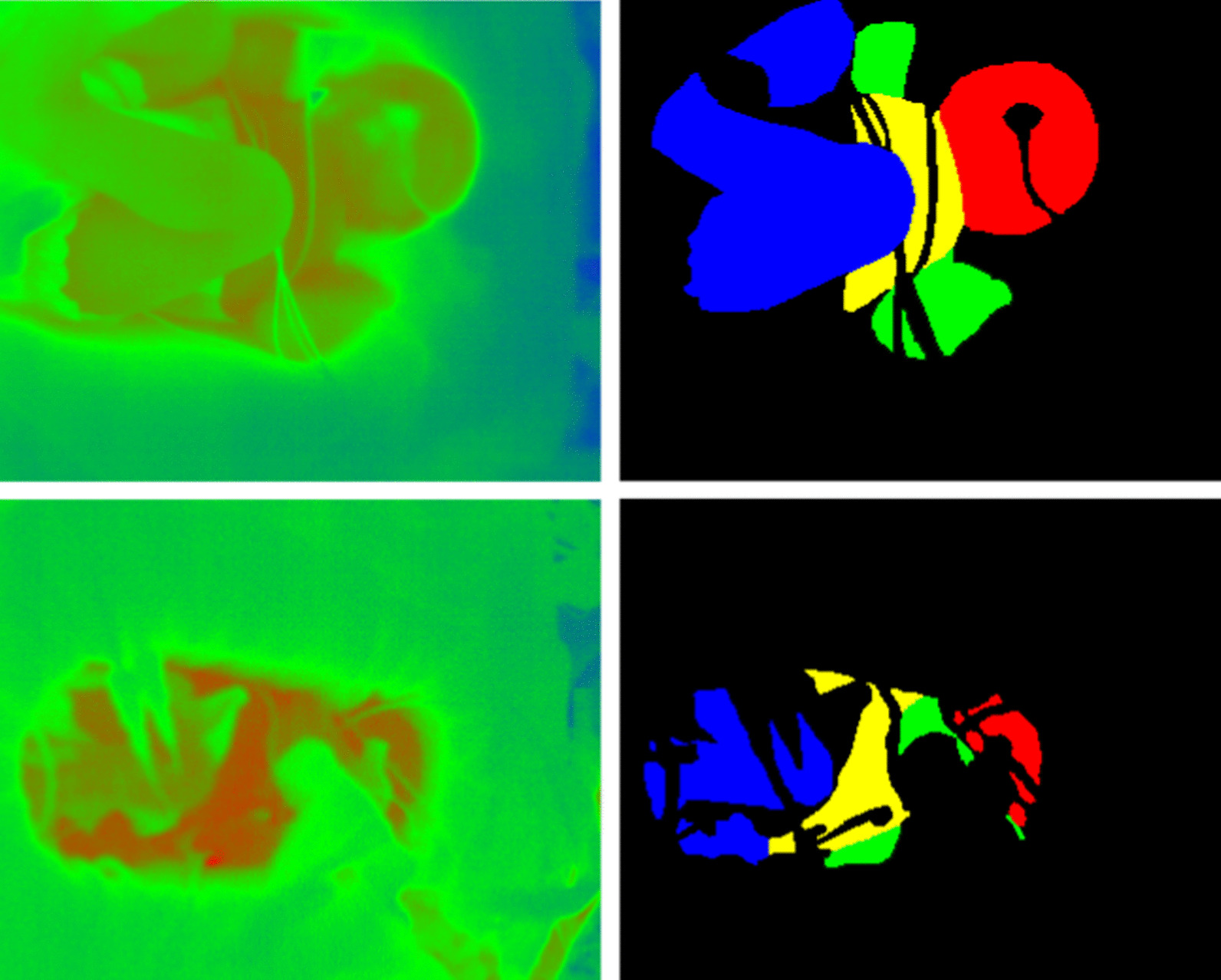
Examples of thermal images and ground truth. The head is shown in red, the body in yellow, the arms in green, the legs in blue, and the other regions in black

A total of 400 images were selected at random from the thermographic images, excluding those taken during treatment or nursing care by medical staff, and the ground truth was generated manually. The pixels of the thermal images were divided into five classes, i.e., head, body, arms, legs, and “other.” The cervical region was defined as the head, and the shoulder region was defined as part of the arm region. In addition, diapers, probes, tubes, respiratory masks, and hair in the images were strictly excluded as non-skin areas. The definition of ground truth was made by a skilled neonatologist, who also checked the generated ground truth, as shown in Fig. 2. Subsequent training and testing were conducted using the generated ground truth.

The network structure was based on U-Net for thermal image segmentation, and we applied the Convolution–Batch Normalization–Rectified Linear Unit (ReLU) (CBR) structure used in ResNet [46]. As U-Net is often the first choice for semantic segmentation of medical images, it was also used in this study as the base architecture and was shown to be suitable for analyzing thermal images of infants. The detailed network structure is shown in Table 2. The total network was a 22-stage fully convolutional network. A number of functions have been proposed to improve the performance of networks, but most have been evaluated only on RGB images, and there have been no reports of evaluation of thermal images. Therefore, Weight Normalization [47], Group Normalization [48], and Flexible Rectified Linear Unit (FReLU) [49], which have already been evaluated on images, were applied to compare their accuracy on thermal images. Weight Normalization was replaced by convolution, Group Normalization by Batch Normalization, and FReLU by ReLU, and all combinations were evaluated. Preliminary experiments were conducted at 2-, 4-, 5-, 8-, and tenfold at the image level, and the experiment was assumed to be conducted at fourfold, where accuracy began to drop. With fourfold cross-validation, the classification accuracy of segmentation and Mean Intersection over Union (mIoU) were used as evaluation metrics. Cross Entropy Loss was used as the loss function. No pre-training was performed.

**Table.2:** Detailed network configuration of U-Net, U-Net GAN Generator, and U-Net GAN + SA Generator

Layers	Output size	U-Net	U-Net GAN + SA
Input	320 × 256 × 1		
Convolution	320 × 256 × 16	3 × 3, 16 d	3 × 3, 16 d
Downscale	160 × 128 × 32	5 × 5, 32 d, CBR 3 × 3, 32 d, CBR	1 × 1, 32 d 7 × 7, 32 d, SA 1 × 1, 32 d
Downscale	80 × 6464	5 × 5, 64 d, CBR 3 × 3, 64 d, CBR	1 × 1, 64 d 7 × 7, 64 d, SA 1 × 1, 64 d
Downscale	40 × 32 × 128	5 × 5, 128 d, CBR 3 × 3, 128 d, CBR	1 × 1, 128 d 7 × 7, 128 d, SA 1 × 1, 128 d
Downscale	20 × 16 × 256	5 × 5, 256 d, CBR 3 × 3, 256 d, CBR	1 × 1, 256 d 7 × 7, 256 d, SA 1 × 1, 256 d
Downscale	10 × 8 × 512	5 × 5, 512 d, CBR 3 × 3, 512 d, CBR	1 × 1, 512 d 7 × 7, 512 d, SA 1 × 1, 512 d
Upscale	20 × 16 × 256	5 × 5, 256 d, CBR 3 × 3, 256 d, CBR	1 × 1, 256 d 7 × 7, 256 d, SA 1 × 1, 256 d
Upscale	40 × 32 × 128	5 × 5, 128 d, CBR 3 × 3, 128 d, CBR	1 × 1, 128 d 7 × 7, 128 d, SA 1 × 1, 128 d
Upscale	80 × 64 × 64	5 × 5, 64 d, CBR 3 × 3, 64 d, CBR	1 × 1, 64 d 7 × 7, 64 d, SA 1 × 1, 64 d
Upscale	160 × 128 × 32	5 × 5, 32 d, CBR 3 × 3, 32 d, CBR	1 × 1, 32 d 7 × 7, 32 d, SA 1 × 1, 32 d
Upscale	320 × 256 × 16	5 × 5, 16 d, CBR 3 × 3, 16 d, CBR	1 × . 1, 16 d 7 × 7, 16 d, SA 1 × 1, 16 d
Convolution	320 × 256 × 1	3 × 3, 1 d	3 × 3, 1 d

**Fig. 3:**
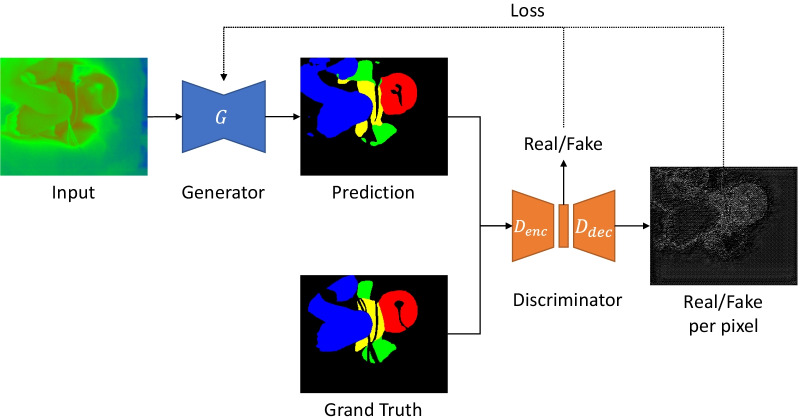
Network diagram of U-Net GAN

Furthermore, based on the network with the highest accuracy in the above comparison, GAN and SA were applied to extend the network, and the accuracy was evaluated again. Here, we extended U-Net GAN [50] proposed by Schonfeld et al., an image generation method that uses U-Net as a Discriminator, and applied it to neonatal thermography. This method optimizes not only the entire image, but also each pixel, resulting in images with fewer errors than traditional GAN. The segmentation system using U-Net GAN is shown in Fig. 3, where $x$ represents the correct data for segmentation and $T$ represents the input thermal image. The output of the generator that performs the segmentation of the thermal image $T$ is denoted by $G\left( T \right)$. The Discriminator has Encoder and Decoder sections, and its output consists of $D_{enc} \left( x \right)$, which predicts the Real/Fake classification of the whole image, and $D_{dec} \left( x \right)$, which predicts the Real/Fake classification of each pixel.

The network with the highest accuracy in the experiments described above is used as the Generator of U-Net GAN. Here, we conducted preliminary experiments, and the Discriminator network was made with four layers of CBR blocks and half the number of channels. Using U-Net GAN, segmentation results were constrained to be similar to the manually generated ground truth, while preserving accuracy and suppressing overfitting. The detailed network structure of U-Net GAN Discriminator is shown in Table 3. The encoder output of the Discriminator is average-pooling of the most downscaled image data of U-net, and the full connect is used to identify the real/fake binary value. Therefore, the encoder output is one data output for one image. The decoder output has the same image size as the input and classifies real/fake on a pixel-by-pixel basis.

**Table.3:** Detailed network configuration of U-Net GAN discriminator and U-Net GAN + SA discriminator

Layers	Output size	U-Net	U-Net GAN + SA
Input	320 × 256 × 1		
Convolution	320 × 256 × 8	3 × 3, 8 d	3 × 3, 8 d
Downscale	160 × 128 × 16	5 × 5, 16 d, CBR 3 × 3, 16 d, CBR	1 × 1, 16 d 7 × 7, 16 d, SA 1 × 1, 16 d
Downscale	80 × 64 × 32	5 × 5, 32 d, CBR 3 × 3, 32 d, CBR	1 × 1, 32 d 77, 32 d, SA 1 × 1, 32 d
Downscale	40 × 32 × 64	5 × 5, 64 d, CBR 3 × 3, 64 d, CBR	1 × 1, 64 d 7 × 7, 64 d, SA 1 × 1, 64 d
Encoder out ($D_{enc} \left( x \right)$)	5	ReLU Average Pooling Linear, 5d	ReLU Average Pooling Linear, 5 d
Upscale	80 × 64 × 32	5 × 5, 32 d, CBR 3 × 3, 32 d, CBR	1 × 1, 32 d 7 × 7, 32 d, SA 1 × 1, 32 d
Upscale	160 × 128 × 16	5 × 5, 16 d, CBR 3 × 3, 16 d, CBR	1 × 1, 16 d 7 × 7, 16 d, SA 1 × 1, 16 d
Upscale	0 × 256 × 8	5 × 5, 8 d, CBR 33, 8 d, CBR	1 × 1, 8 d 7 × 7, 8 d, SA 1 × 1, 8 d
Convolution ($D_{dec} \left( x \right)$)	320 × 256 × 2	3 × 3, 2 d	3 × 3, 2 d

In addition to U-Net GAN, SA was used to improve performance. Unlike RGB images, thermal images represent single-channel data of temperature only, and the relationships between the temperatures are important for the analysis. Therefore, application of the SA module to the network will make it possible to evaluate not only the spatial relations but also the appearance patterns of heat and feature intensities, which will enable more detailed analysis. The structure of the network with incorporation of the SA module into U-Net GAN (U-Net GAN + SA) is shown in Table 2. The number of channels remains unchanged, although the depth of the network is increased due to the bottleneck structure. The loss function of the Discriminator, ${\mathcal{L}}_{D}$, was calculated using Eq. 1:

1 $${\mathcal{L}}_{D} = {\mathcal{L}}_{{D_{enc} }} + {\mathcal{L}}_{{D_{dec} }} + {\mathcal{L}}_{{D_{dec} }}^{cons}$$

where ${\mathcal{L}}_{{D_{enc} }} ,{\mathcal{L}}_{{D_{dec} }}$, and ${\mathcal{L}}_{{D_{dec} }}^{cons}$ are the Encoder Loss, Decoder Loss, and Consistency Loss of the Discriminator, respectively, and are expressed in Eqs. 2–4:

2 $${\mathcal{L}}_{{D_{enc} }} = - {\mathbb{E}}_{x} \left[ {{\text{log}}D_{enc} \left( x \right)} \right] - {\mathbb{E}}_{T} \left[ {\log \left( {1 - D_{enc} \left( {G\left( T \right)} \right)} \right)} \right]$$

3 $${\mathcal{L}}_{dec} = - {\mathbb{E}}_{x} \left[ {\frac{{\mathop \sum \nolimits_{{{\text{i}},{\text{j}}}} \log \left[ {D_{dec} \left( x \right)} \right]_{i,j} }}{width*height}} \right] - {\mathbb{E}}_{T} \left[ {\frac{{\mathop \sum \nolimits_{{{\text{i}},{\text{j}}}} \log \left( {1 - \left[ {D_{dec} \left( {G\left( T \right)} \right]_{i,j} } \right])} \right)}}{width*height}} \right]$$

4 $${\mathcal{L}}_{{D_{dec} }}^{cons} = ||D_{dec} ({\text{mix}}\left( {x, G\left( T \right), {\text{M}}} \right) - {\text{mix}}\left( {D_{dec} \left( x \right), D_{dec} \left( {G\left( T \right)} \right), {\text{M}}} \right)||^{2}$$

where ${\text{mix}}\left( {x_{1} , x_{2} , {\text{M}}} \right)$ is the CutMix function [51], which mixes $x_{1}$ and $x_{2}$ according to the mask ${\text{M}}$, and $width$ and $height$ are the width and height of the image, respectively. The loss is given by ${\mathcal{L}}_{{D_{enc} }}$ to correctly predict the Real/Fake classification of the whole image, and by ${\mathcal{L}}_{dec}$ to correctly predict the Real/Fake classification of each pixel. Consistency Loss also improves the stability of the Discriminator’s prediction by placing constraints on the CutMix of $D_{dec} \left( x \right)$ and $D_{dec} \left( {G\left( T \right)} \right)$ and the prediction results of the CutMix of $x$ and $G\left( T \right)$ to be the same. The loss function, ${\mathcal{L}}_{G}$, of the generator is also shown in Eq. 5:

5 $${\mathcal{L}}_{G} = - {\mathbb{E}}_{T} \left[ {\log D_{enc} \left( {G\left( T \right)} \right) + \frac{{\mathop \sum \nolimits_{i,j} \log \left[ {D_{dec} \left( {G\left( T \right)} \right)} \right]_{i,j} }}{{\text{width*height}}}} \right] + \lambda \cdot \frac{{\mathop \sum \nolimits_{i,j} CrossEntropy\left( {x, G\left( T \right)} \right)}}{width*height}$$

The first term represents the loss of the Discriminator and constrains segmentation to be similar to the ground truth. $CrossEntropy\left( {x_{1} , x_{2} } \right)$ represents the Cross Entropy Loss, and $\lambda$ is a variable that balances the first and second terms; in this paper, $\lambda = 0.1$.

As in the previous experiment, fourfold cross-validation was performed to evaluate U-Net GAN and U-Net GAN + SA. In addition to classification accuracy and mIoU, a Confusion Matrix including U-Net was used as an evaluation metric.

For training, a PC with an AMD Ryzen 7 3700X CPU, 64 GB of memory, and a GeForce RTX 3090 GPGPU running Windows 10 was used. We used Python 3.7 as the programming language and Pytorch 1.1 was used as a deep learning package. The optimal values of learning parameters (i.e., network depth, number of channels per layer, batch size, learning rate) were determined through a preliminary experiment. The number of training epochs was determined before the model began overfitting. The parameters used for training are shown in Table 4. For Augmentation, we performed a vertical flip of the image and added random noise to each pixel. AMSGrad [52] was used as the optimizer.

**Table.4:** Parameters used for training

Parameter	Net	U-Net GAN	U-Net GAN + SA
Learning rate	0.01	0.01 (generator)	0.01 (generator)
		1e−4 (discriminator)	1e−4 (discriminator)
Batch size	75	30	12
Epoch	200	100	100

Statistical analyses were conducted to compare the accuracy between the methods. The Steel–Dwass test was applied as a nonparametric multiple comparison test. All analyses were performed using JMP 15 statistical software. For a detailed evaluation of segmentation performance, the Hausdorff distance and IoU for each region were calculated.

## Results

The accuracy of segmentation using U-Net was evaluated and the results are shown in Table 5. Even standard U-Net showed very high segmentation accuracy with a validation accuracy of 91.3% (SD 0.04%) and mIoU of 57.8% (SD 0.15%). FReLU showed improvements of 0.6% (SD 0.04%) in accuracy and 3.1% (SD 0.16%) in mIoU, while Group Normalization showed improvements of 0.9% (SD 0.04%) in accuracy and 4.4% (SD 0.14%) in mIoU. However, Normalized Convolution decreased the accuracy by 0.2% (SD 0.05%), but improved the mIoU by 3.1% (SD 0.15%). The best results were obtained with the combined application of FReLU and Group Normalization showing 92.9% (SD 0.04%) accuracy and mIoU of 64.5% (SD 0.15%).

**Table.5:** Segmentation performance using U-Net with and without normalized convolution, FReLU, and group normalization

Normalized convolution	FReLU	Group normalization	Accuracy (%)	SD (%)	mIoU (%)	SD (%)
			91.3	0.04	57.8	0.15
✓			91.1	0.05	60.9	0.15
	✓		91.9	0.04	60.9	0.16
		✓	92.2	0.04	62.2	0.14
✓	✓		91.4	0.05	60.7	0.15
✓		✓	92.4	0.04	63.8	0.13
	✓	✓	92.9	0.04	64.5	0.15
✓	✓	✓	92.4	0.04	62.9	0.15

**Fig. 4:**
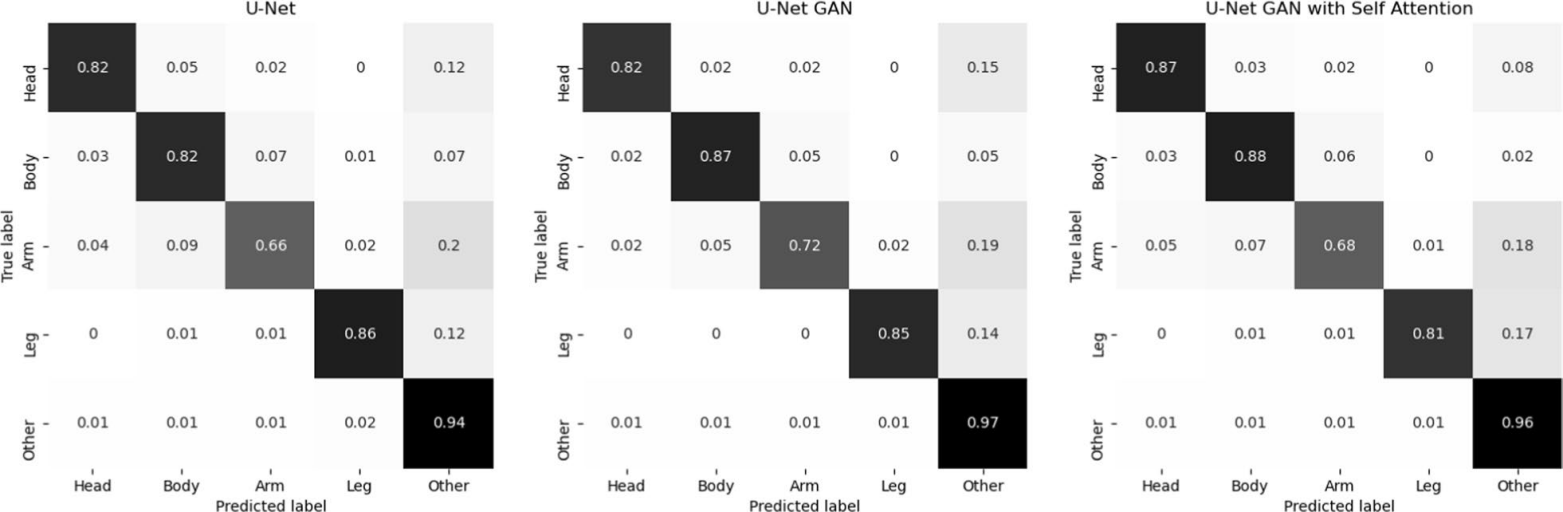
Confusion matrices of U-Net, U-Net GAN, and U-Net GAN + SA

U-Net GAN and U-Net GAN + SA showed validation accuracy of 93.3% (SD 0.03%) and 93.5% (SD 0.04%), representing improvements of 0.7% and 0.9%, respectively, and mIoU of 66.9% (SD 0.13%) and 70.4% (SD 0.13%), representing improvements of 2.4% and 5.9%, respectively, compared to the best results of U-Net (Table 6). Finally, the confusion matrices for U-Net, U-Net GAN, and U-Net GAN + SA are shown in Fig. 4. For each network, the accuracy was 82%, 82%, and 87% for head, 82%, 87%, and 88% for body, 66%, 72%, and 68% for arms, 86%, 85%, and 81% for legs, and 94%, 97%, and 96% for other, respectively. The results of the Steel–Dwass test are shown in Table 7. Significant differences were found between several methods. The results of the Hausdorff distance and IoU for each region are shown in Tables 8 and 9, respectively.

**Table.6:** Segmentation performance of U-Net, U-Net GAN, and U-Net GAN + SA

Network	Accuracy (%)	SD (%)	mIoU (%)	SD (%)
U-Net	92.9	0.04	64.5	0.15
U-Net GAN	93.3	0.03	66.9	0.13
U-Net GAN + SA	93.5	0.03	70.4	0.13

**Table.7:**
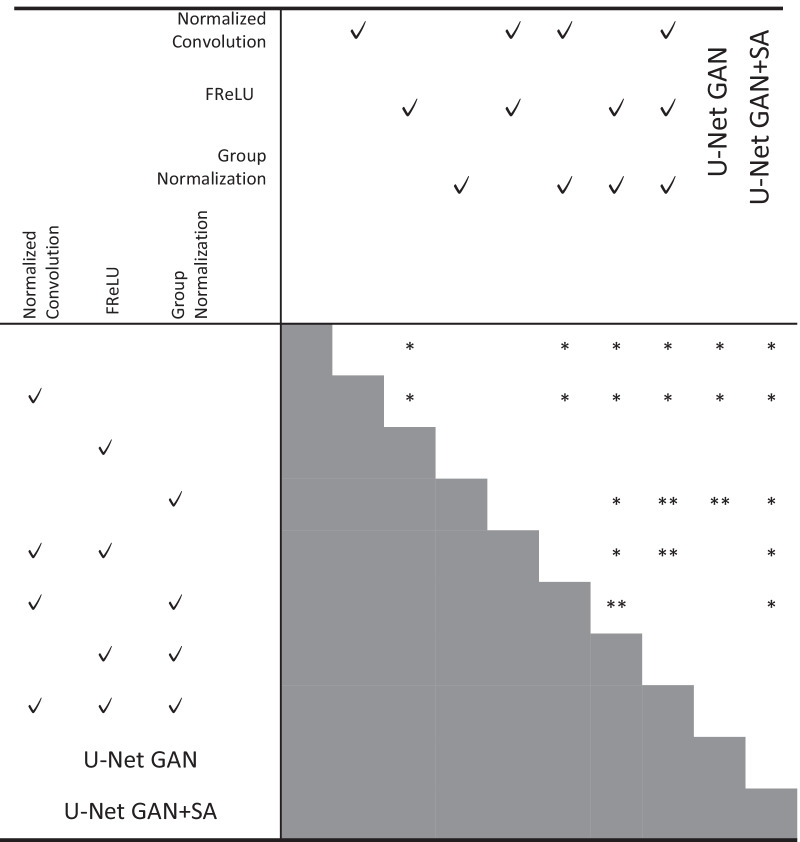
Significant differences between the proposed methods

*p < 0.01, **p < 0.05

**Table.8:** Hausdorff distance for each region

	Head		Body		Arm		Leg		Other		All (w/o other)	
	Mean	SD	Mean	SD	Mean	SD	Mean	SD	Mean	SD	Mean	SD
UNet	34.6	25.3	38.1	29.5	59.2	37.7	43.4	40.0	26.7	9.3	43.9	34.8
Normalized convolution	33.0	23.6	31.5	26.2	55.7	35.6	46.5	50.7	26.5	8.9	41.5	36.4
FReLU	31.2	21.3	31.2	22.6	58.4	38.0	42.8	40.0	25.5	9.1	40.8	33.3
Group normalization	30.3	18.2	30.1	21.9	63.4	40.0	50.7	48.6	25.8	9.5	43.4	36.9
Normalized convolution FReLU	27.8	18.9	31.1	25.6	57.2	37.1	47.9	47.5	25.5	9.2	40.8	35.7
Normalized convolution group normalization	30.4	21.3	30.0	20.6	64.9	34.1	52.9	50.0	26.7	9.9	44.3	36.3
FReLU group normalization	27.5	17.8	25.2	20.2	48.7	32.8	38.6	38.3	25.4	8.8	34.9	29.8
ALL	26.8	17.2	28.5	22.4	53.9	36.9	48.5	49.9	25.3	9.9	39.1	35.5
U-Net GAN	27.4	19.7	26.7	22.7	49.3	34.1	39.0	42.7	24.5	9.1	35.5	32.1
U-Net GAN + SA	27.1	17.7	26.7	22.7	46.3	32.6	41.4	42.1	23.7	9.4	35.2	31.1

**Table.9:** IoU for each region

	Head		Body		Arm		Leg		Other		All	
	IoU (%)	SD (%)	IoU (%)	SD (%)	IoU (%)	SD (%)	IoU (%)	SD (%)	IoU (%)	SD (%)	IoU (%)	SD (%)
UNet	50.8	0.16	52.1	0.16	41.6	0.17	53.5	0.23	91.1	0.03	57.8	0.15
Normalized convolution	57.5	0.14	48.5	0.15	47.4	0.15	59.8	0.23	91.5	0.03	60.9	0.14
FReLU	54.8	0.17	56.6	0.16	44.3	0.18	57.1	0.26	91.8	0.03	60.9	0.16
Group normalization	56.4	0.16	60.1	0.15	43.1	0.16	58.6	0.24	93.0	0.03	62.2	0.15
Normalized convolution FReLU	55.1	0.14	57.3	0.14	41.4	0.13	57.6	0.21	92.2	0.03	60.7	0.13
Normalized convolution group normalization	58.0	0.15	61.2	0.16	47.3	0.17	60.2	0.24	92.4	0.03	63.8	0.15
FReLU group normalization	59.2	0.16	62.3	0.15	47.7	0.17	61.4	0.23	92.0	0.03	64.5	0.15
ALL	58.2	0.15	59.3	0.15	47.9	0.16	58.0	0.25	91.3	0.03	62.9	0.15
U-Net GAN	61.5	0.14	64.3	0.14	49.1	0.15	66.4	0.2	93.4	0.03	66.9	0.13
U-Net GAN + SA	64.8	0.14	67.9	0.14	57.9	0.14	67.6	0.2	93.6	0.02	70.4	0.13

## Discussion

All of the methods examined here showed highly accurate classification performance. FReLU and Group Normalization improved the classification accuracy and mIoU of U-Net, which was considered to be due to the improved representativeness of the network. Group Normalization shows that normalization within the channels of the network is more effective than Batch Normalization in this problem. This was because the input data consisted only of temperature information with similar backgrounds, so there were many regions with similar values, and Batch Normalization may have the effect of over-averaging the data. On the other hand, Normalized Convolution showed a decrease in accuracy but an improvement in mIoU. Depending on the location of the thermal imaging camera and the view angle, the “other” region had 13–23 times more pixels than the “infant” region. Thus, Normalized Convolution may decrease the number of missed skin regions, but increase the percentage of false positive identification of other regions as skin regions. The application of U-Net with FReLU and Group Normalization showed 1.6% better accuracy and 6.7% better mIoU than ReLU and Batch Normalization. These results confirmed that the combined use of these tools resulted in significant improvements, especially in mIoU.

Using the network with FReLU and Group Normalization applied to U-Net as a baseline, U-Net GAN and U-Net GAN + SA were confirmed to show beneficial effects.

Compared to the accuracy of U-Net of 92.9%, U-Net GAN showed a 0.4% improvement in accuracy and 2.4% improvement in mIoU, and U-Net GAN + SA improved accuracy by 0.6% and mIoU by 5.9%.

The results of the Steel–Dwass test showed significant differences between several methods. In particular, FReLU alone showed a significant performance improvement. There was no significant difference between FReLU and U-Net GAN + SA, thus confirming the effectiveness of FReLU. U-Net GAN + SA showed significant differences in many cases compared to the other methods, confirming that it is a powerful method. However, there were no significant differences between the four sets of results: FReLU with Group Normalization, FReLU with Group Normalization and Normalized Convolution, U-Net GAN, and U-Net GAN + SA. This suggests that the performance improvement may be approaching its limit.

Similar results were obtained with Hausdorff distance. FReLU with Group Normalization, U-Net GAN, and U-Net GAN + SA performed better than the other methods in almost all regions, and the SD was also lower. In all methods, the Hausdorff distance was larger for the arms and legs than for the head and body. In IoU, Other was the highest in all methods, which may have been due to the lower temperature in the Other region compared to the neonate, thus making segmentation easier. U-Net GAN + SA showed better results for infant region segmentation. SA was also effective in Semantic Segmentation of thermal images.

**Fig. 5:**
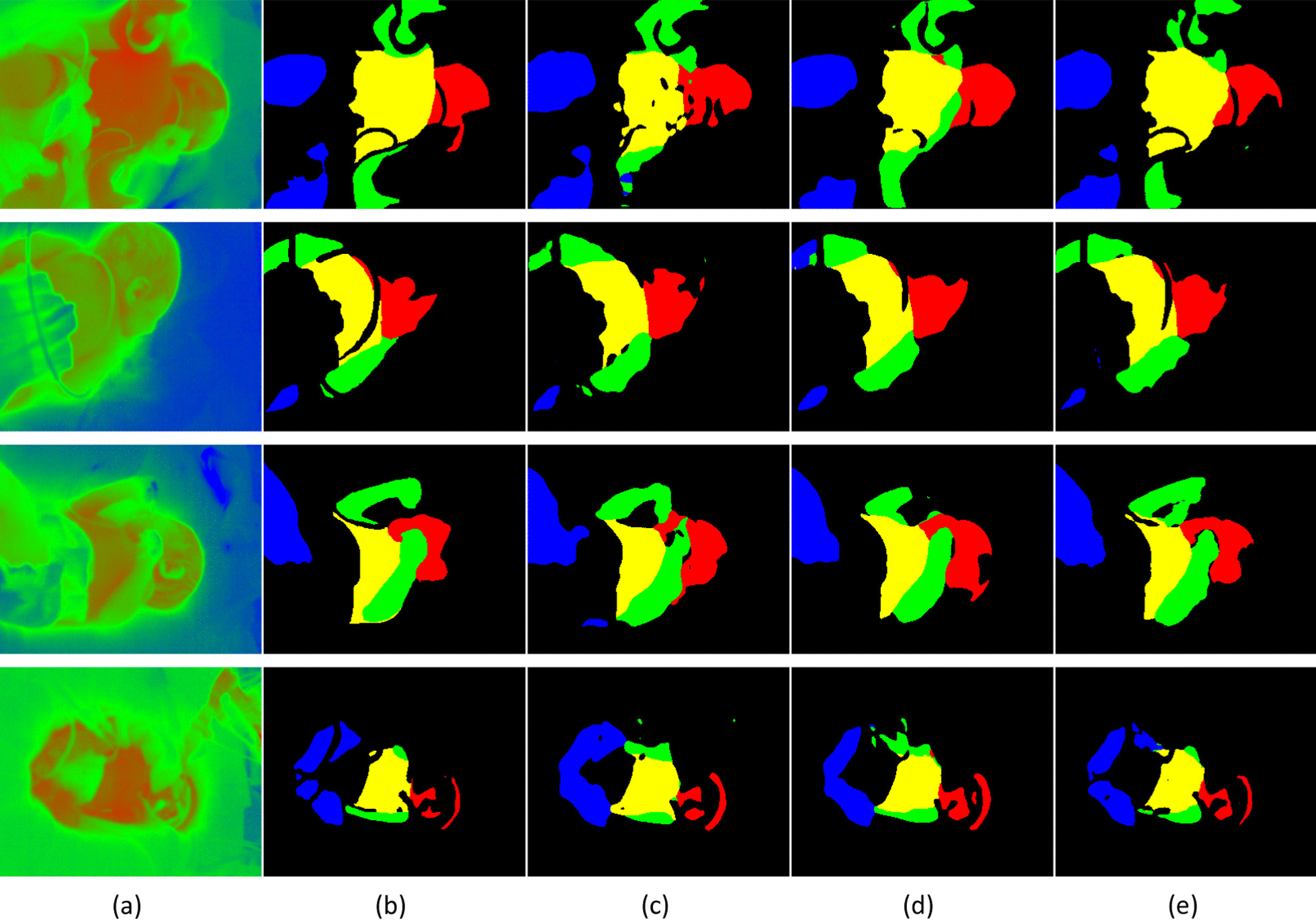
Examples of the differences in segmentation results between U-Net, U-Net GAN, and U-Net GAN + SA. **a** Input. **b** Ground truth. **c** U-Net. **d** U-Net GAN. **e** U-Net GAN + SA

U-Net GAN is optimized by combining multiple loss functions. The Discriminator classifies the manually generated ground truth and the results of U-Net segmentation, and in addition to the conventional GAN evaluation on a per-image basis, it also evaluates and feeds back the results on a per-pixel basis. This yields not only higher performance than normal U-Net, but is also visually closer to the manually obtained ground truth. The accuracy was further improved in U-Net GAN + SA by changing the Convolution to SA. SA, which strictly evaluates the relationship between pixels, was considered to be effective as temperature images have lower value variation and dimensionality compared to RGB images. The temperature image, ground truth, and images obtained by segmentation using U-Net, U-Net GAN, and U-Net GAN + SA are shown in Fig. 5. The results of all methods showed high accuracy, but the features differed between methods. U-Net segmented the images with smooth boundaries. On the other hand, it misdetected thin regions, such as cables on the body surface, resulting in finely over-segmented regions. U-Net GAN yielded a smoother segmentation shape and unnatural segmentation was prevented, and U-Net GAN + SA successfully excluded fine non-skin areas, such as cables and the shapes near the boundaries of the segmented areas followed the edges of the temperature information. These results were attributed to the strict evaluation of temperature relationships by SA, resulting in detailed semantics.

The confusion matrix shown in Fig. 4 indicated that the detection accuracy of each region differed between methods. U-Net GAN + SA showed 5% higher detection accuracy for the head than the other methods. For the body, U-Net GAN and U-Net GAN + SA showed 5%–6% higher accuracy than U-Net. For the arms, U-Net GAN was 4–6% more accurate than the other methods, and for the legs, U-Net was 1–5% more accurate than the other methods. U-Net GAN showed 1–3% higher accuracy for the other regions than the other methods. The features of the resulting segmented images differed according to the method used, although the numerical differences were small. U-Net GAN + SA predicted the skin region of the infant as “other” less frequently than the other methods, which was due to the strict evaluation of pixel-by-pixel temperature relationships by SA. The accuracy of U-Net GAN + SA was higher for the head and body compared to the other methods, while it showed lower accuracy for the arm and leg regions due to an increase in the number of cases where they were incorrectly detected as other skin regions. This was because the arms and legs have more variations in shape and positional relationships than the head and body, and strictly evaluating the pixel-by-pixel relationships leads to incorrect predictions. Therefore, additional training data and further augmentation are considered necessary for U-Net GAN + SA to detect arms and legs more accurately. U-Net and U-Net GAN tended to have slightly lower accuracy than U-Net GAN + SA. However, SA requires a great deal of processing and large amounts of memory, so it is important to consider the device to be used and select the optimal method to be applied. In medical applications, it is not necessary to evaluate the temperature of areas other than the skin, and therefore U-Net GAN + SA is considered to be effective. However, further improvements are needed for regions where the shape and positional relationships may vary, such as the arms and legs, as the system showed degradation of performance in such areas.

The application of this method in clinical settings will enable continuous monitoring of temperature in each region of the body. Further studies are required to confirm the effectiveness of this method in managing the body temperature of infants and analyzing various diseases.

Further studies are required to evaluate the accuracy of measuring the body temperature of infants using our method. The segmentation accuracy was evaluated, but the impact of this accuracy on the temperature measurement is not yet clear. Furthermore, large amounts of clinical data will be collected and analyzed using the results obtained with this method to study the ability to predict diseases and other conditions. In this process, the accuracy required for segmentation will be clarified. It will be necessary to examine these issues through clinical application in future studies.

## Conclusion

A U-Net-based network was confirmed to be able to segment the skin area on thermographic thermal images of infants with high accuracy. FReLU and Group Normalization were confirmed to be effective for thermal image segmentation. GAN was also shown to improve the segmentation accuracy, and SA achieved fine segmentation even on thermal images with few features. These tools contributed to the improvement of mIoU, and U-Net GAN + SA showed a significant performance improvement over standard U-Net.

## Data Availability

The datasets generated and analyzed during the present study are not publicly available due to participant privacy, but are available from the corresponding author on reasonable request.

## Abbreviations

CBR structure: Convolution–batch normalization–ReLU structure
CT: Computed tomography
FReLU: Flexible rectified linear unit
GAN: Generative adversarial network
MARSI: Medical adhesive-related skin injuries
mIoU: Mean intersection over union
MRI: Magnetic resonance imaging
NHMC: Nagasaki harbor medical center
NEC: Necrotizing enterocolitis
ReLU: Rectified linear unit
ROI: Region of interest
SA: Self-attention
SD: Standard deviation
U-Net GAN: U-Net generative adversarial network
U-Net GAN + SA: Self-attention module in U-Net GAN
VLBW: Very low birth weight
WHO: World Health Organization
